# From Dietary Cholesterol to Blood Cholesterol

**DOI:** 10.3390/nu15143086

**Published:** 2023-07-10

**Authors:** Frans Stellaard

**Affiliations:** 1 Department of Nutrition and Movement Sciences, NUTRIM (School of Nutrition and Translational Research in Metabolism), Maastricht University Medical Center, P.O. Box 5800 Maastricht, The Netherlands; f.stellaard@maastrichtuniversity.nl; Tel.: +31-652579392; 2 Institute of Clinical Chemistry and Clinical Pharmacology, University Hospital Bonn, Venusberg-Campus 1, 53127 Bonn, Germany

The *Nutrients*’ Special Issue “From dietary cholesterol to blood cholesterol” aims to supply existing knowledge and novel new research data about human cholesterol (C) fluxes. The Special Issue contains eight review papers with updated knowledge concerning the complex endogenous and exogenous aspects of C metabolism as well as seven papers describing novel new information, all adding to a better understanding of C homeostasis.

To study the association between dietary C and blood C concentrations, one needs to understand the whole-body C metabolism. The general idea, that an enhanced intake of dietary C leads to enhanced C concentrations in blood, is far too simplified. Analyzing epidemiological studies and meta-analyses, Fernandez and Murillo [1] showed the lack in an association between dietary C and blood C. The explanation for this may be found in the regulatory mechanisms within C metabolism. The physiological lipid fluxes and C homeostasis were described by Stellaard et al. [2]. The intestinal flux of C does not solely consist of dietary C but is dominated by biliary C derived from hepatic secretion, temporary storage in the gallbladder and secretion from the gallbladder following neurohormonal stimuli. Furthermore, under physiological conditions, the intestinal fractional absorption rate (FAR) of C is highly variable, ranging from 20 to 80% [3,4]. Thus, only a high dietary C intake combined with a high biliary C secretion rate and a high C FAR may lead to a clear, high C input into the endogenous C. The endogenous C pool is not only determined by C absorption but also by endogenous C synthesis. Under physiological, dietary C intake conditions, C FAR and C synthesis are negatively associated, indicating a reduced C synthesis when C FAR is high and an induced C synthesis when C FAR is low [5]. Hepatic C metabolism determines C homeostasis, coordinating several C fluxes entering the liver as high density lipoprotein (HDL-C), low density lipoprotein (LDL-C), chylomicron remnant (CMR-C), hepatic C synthesis, and leaving the liver as very low-density lipoprotein (VLDL-C), biliary C secretion, and synthesis of bile acids (BAs). Thus, C synthesis and C FAR are the major determinants affecting the C input into the endogenous C pool when dietary C intake is not enhanced [2].

It is generally expected that enhanced C synthesis and C FAR are determinants of the serum C concentration and, predominantly, the LDL-C concentration. However, Stellaard et al. [6] demonstrated a lack in correlation between serum LDL-C and C synthesis or C FAR in healthy subjects, when measured directly or when approached by respective marker technologies. It is concluded that the effects of C synthesis and C FAR are diluted by the effects of the other mentioned hepatic C fluxes. Elevated serum LDL-C is considered the most predictive atherogenic criterium to express enhanced risk for CVD and LDL-C lowering therapy is generally started [7]. The LDL-C lowering target is determined by the additional risk factors such as smoking, obesity, and diabetes. Recently, it has become evident that cholesterol in LDL is not the only atherogenic factor. The C present in more triglyceride-rich lipoproteins (TRL) such as VLDL remnants, IDL remnants, and chylomicron remnants (CMR) exhibit high atherogenic potential [8]. Lütjohann et al. [9] described the potentials and limitations of determining the TRL-C with rapid, commercially available analytical techniques applicable in routine clinical diagnostic laboratories. The treatment of enhanced serum TRL-C requires the development of alternative treatment methods.

Mashnafi et al. [10] studied the effect of a low calorie diet on C synthesis and C FAR in obese male subjects using the marker technology. They concluded that diet induced weight reduction and led to reduced C synthesis and enhanced C absorption in conjunction with reduced total C and LDL-C in serum. Unfortunately, the authors did not measure the dietary C intake. A low calorie diet may include a low C intake and possibly an induced C FAR. Lytle et al. [11] studied the effects of low-fat ground beef and high-fat ground beef on the voluntary adaptation of the food composition, on the serum lipoprotein levels, and on lipoprotein composition. Interestingly, the overall fat intake in the low-fat intervention period was lower than at entry. The overall fat intake during the high-fat intervention period was only slightly higher than at entry. These effects are explained by the pan broiling of the beef patties, which led to significant fat loss. Both intervention diets contained less cholesterol compared with the intake values at entry. In both intervention periods, the serum LDL-C concentrations were lower than at entry. Unfortunately, no data were available for the daily flux of biliary C, nor for the C FAR. This study shows that alterations in dietary food composition are not automatically translated into expected, altered serum lipid profiles. Food preparation, food adaptation, and endogenous responses to the dietary changes affect the final results. Houttu et al. [12] observed important effects in three patients consuming extreme high-fat, low-carbohydrate diets. In two patients, the protein and fat intake expressed 37 and 61 E-% and carbohydrate intake less than 3 E-%. With 16, 9, and 12 mmol/L, the LDL-C concentrations in the three patients were at the level of homozygous familial hypercholesterolemic patients. Interestingly, dietary modifications towards reduction in animal fat intake clearly reduced their serum LDL-C values. Apparently, extreme high-animal fat diets do enhance serum LDL-C and the introduction of extreme reductions in animal fat intake do reduce LDL-C.

Due to required invasive methodologies, metabolic studies of lipid metabolism cannot be performed in humans. Experimental animals are used instead with a preference for mice. However, lipoprotein metabolism and lipid metabolism are different between humans and natural mice. Therefore, mice with humanized lipid metabolism have been developed. The latest model is described as APOE*3-Leiden.CETP mice, as described and applied by Paalvast et al. [13]. The authors analyzed the effect of a high-fat, high-C diet in the responding mice establishing an increased serum TG response and in the non-responding mice. Their main conclusions were that, in contrast to the responding animals, the non-responding animals developed a fat malabsorption possibly caused by a decreased production of BAs. The authors suggested that the variation in BA homeostasis may in part drive the phenotypic variation in the APOE*3-Leiden.CETP mice.

An important line of recent research on C metabolism is focused on the role of phytosterols. Phytosterols are present in plants and enter the body only via the diet. The majority are present as sitosterol and campesterol. These compounds have a similar chemical structure as C, differing in an additional methyl-group in the side chain. Phytosterols are transported similarly compared to C, with both transported in the intestine as in lipoproteins. However, the FARs of phytosterols are much lower than the C FAR, i.e., less than 10%. One research topic deals with the fact that a high phytosterol concentration in serum is expressed as a reflection of high C absorption. The ratio between plant sterol concentrations to total C in fasting serum is used as a surrogate marker for C absorption. Otherwise, phytosterol absorption has been shown to compete with C absorption at the level of uptake into micelles transporting sterols to the site of absorption. This has led to the development of food supplements containing phytosterols and/or stanols in order to reduce C absorption and lower serum LDL-C and therewith the enhanced risk for cardiovascular disease (CVD). However, the potential risk of elevated serum phytosterol concentrations for CVD development has been postulated in the literature. Windler et al. [14] discussed the pros and cons of phytosterol treatment and concluded that the regular dose of the supplement must be considered too low to introduce a significant risk for cardiovascular disease in the general population.

Non-C sterols represent a class of compounds combining C precursors in the C synthesis pathway (lanosterol, lathosterol, desmosterol, stigmasterol). The ratio between their concentrations and the total C concentration in fasting serum are considered markers for C synthesis. Van Brakel et al. [15] described an unexpected potential function of non-C sterols. In up to two year-old children, the authors were able to show that the odds of eczema were lower with higher non-C sterols in breast milk. The odds of allergic sensitization at age two were lower with higher concentrations of campesterol in breast milk.

BAs are hepatic metabolites of C and have many distinct functionalities. Synthesis of BAs is a major factor in the removal of endogenous C. Otherwise, they serve to produce mixed micelles and transport lipophilic compounds such as C and triglycerides through the intestine and C in bile. In recent years, BAs have been identified as signaling compounds in the regulation of many processes. At first, absorbing circulating BAs regulate the hepatic BA synthesis rate and control the rate of gallbladder relaxation. Secondly, they are involved in the regulation of many independent metabolic processes. These processes and mechanisms of action, involving mainly the farnesoid X receptor (FXR) and enterokine fibroblast growth factor 19 (FGF19), are described in detail by Di Ciaula et al. [16].

Disturbances in the gut microbiota have been repeatedly associated with CVD, including atherosclerosis and hypertension. Short-chain fatty acids, trimethylamine-N-oxide, and BAs have been identified as causal factors. The functionality of the BA pool is dependent on its composition. Different BAs have one to three hydroxyl groups in different positions. This determines the hydrophobicity of the individual BA pool. Per day, 30 to 50% of the primary BA pools (cholic acid and chenodeoxycholic acid) are malabsorbed and converted to secondary BAs. The bacterial products formed are determined by the gut microbial load and composition. The effects of microbial BA modulations on the etiology of cardiovascular diseases were discussed by Yntema et al. [17].

As mentioned, seven C fluxes are affecting the hepatic C homeostasis. The regulation of these fluxes is a critical factor in the establishment of the hepatic C pool and the flux of LDL-C being removed from the blood. The size of the hepatic C pool affects the regulation of hepatic C synthesis and the synthesis of the LDL receptor affecting the hepatic extraction of serum LDL. However, little is known about the regulation of the other C fluxes. Interestingly, in this Special Issue, two contributions point to the potential role of microRNAs (miRNAs) in hepatic regulation. miRNAs are small, single-stranded, non-coding RNA molecules containing 21 to 23 nucleotides. Konings et al. [18] performed a systematic review of the literature describing the involvement of miRNAs in the development of non-alcoholic fatty liver disease (NAFLD) associated with C metabolism due to accumulation of hepatic free C. They identified miR122, miR34a, miR21, and miR132 as potential candidates to play a role in the development of NAFLD via effects on cholesterol metabolism. Sidorkiewicz [19] contributed a review paper focusing on miR33 and described evidence for the involvement of miR33 in the regulation of reversed C transport via HDL production. A role for miR33 is also suggested for bile formation. Thus, microRNA research may clarify hepatic C homeostasis in the near future and offer new tools for treatment.

A field not covered so far is C metabolism in the brain. C cannot pass the blood–brain barrier. In the brain, C is synthesized mainly in the astrocytes and metabolized to 24S-C in the neurons. A disturbed brain C metabolism is associated with Alzheimer’s disease (AD) and stimulation of cholesterol turnover in the brain was found to ameliorate the development of cognitive decline in AD mice. Martens et al. [20] described the upregulation of C efflux genes and downregulation of lipogenesis genes by incubation with lipophilic extracts of six European brown seaweed species. Their results confirm the described prevention of disease progression in AD mice by the Asian brown seaweed species *Sargassum fusiforme* [21]. Thus, supplementation with extracts of European brown seaweed species may become a strategy in the prevention and/or treatment of neurodegenerative diseases and possibly cardiometabolic and inflammatory diseases.
